# Impact of digitization on carbon productivity: an empirical analysis of 136 countries

**DOI:** 10.1038/s41598-024-55848-2

**Published:** 2024-03-01

**Authors:** Hongna Yu, Huan Liu

**Affiliations:** Harbin University of Commerce, Harbin, 150028 Heilongjiang People’s Republic of China

**Keywords:** Digitalization, Carbon productivity, Technological innovation, Income inequality, Ecology, Environmental sciences, Environmental social sciences

## Abstract

Enhancing carbon productivity (CP) is key to achieving carbon reduction goals while maintaining economic growth. Digital technology plays a significant role in improving CP. Based on panel data from 136 countries worldwide from 2000 to 2020, this study empirically examines the impact of digitalization on CP and its mechanisms using fixed-effects and mediation models. The conclusions are as follows: (1) Overall, digitalization significantly enhances CP. (2) In terms of the mechanism, digitalization primarily improves CP through technological innovation and mitigating income inequality. (3) In terms of the quantile regression results, as the quantile level of CP increases, the promoting effect of digitalization on CP gradually strengthens. (4) From the perspective of heterogeneity among regions, income levels and human capital levels, digitalization has the greatest promotion effect on carbon productivity in European countries, high-income countries and high human capital countries. This study provides a reference for policymakers worldwide to use digital technology in achieving carbon emission reduction targets.

## Introduction

With the economy experiencing rapid growth, the demand for electricity, oil, natural gas, and other energy sources has surged, leading to a substantial increase in CO_2_ emissions^1^. According to the World Bank, the global carbon emissions have increased from 22.5 billion tons in 1998 to 40.5 billion tons in 2022. The massive emission of CO_2_ has caused global warming, which seriously endangers human health and economic efficiency^2^. To address this issue, nearly 200 countries sign the “Paris Agreement” during the 21st United Nations Climate Change Conference. They also formulate and announce their respective national carbon emission reduction targets^3^. Since most countries in the world are facing the pressure of economic development, the realization of carbon emission reduction targets cannot be achieved at the expense of national economies^4^. Therefore, how to reduce CO_2_ emissions while maintaining economic growth is an urgent problem for all countries in the world^5^. Improving CP is the key to solve this problem.

CP refers to the amount of carbon dioxide emissions produced in the process of producing a certain economic output^6^. In order to improve CP, scholars have begun to explore the impact of factors such as economy^7^, technology^8,9^, population^10^, transportation^11^, and policy^12,13^ on CP. However, in the process of stable economic development, the marginal impact of the above factors on CP has not changed significantly. Digitalization is an economic form. In this form, digital technology and knowledge information become the main production factors, used to optimize the process of economic activities and improve production efficiency^14^. With the development and application of digital technology, the growth trend of CP has risen sharply. On one hand, digital technologies, such as the Internet of Things (IoT) and advanced sensors, can furnish enterprises with real-time equipment data. Utilizing real-time data, enterprises can monitor the operational status of their equipment, promptly identify and address inefficiencies, leading to improved energy efficiency^15^. Meanwhile, digitization can also drive the development of environmentally friendly vehicles such as electric cars and self-driving vehicles, thus reducing CO_2_ emissions. On the other hand, digitization can improve CP through technological innovation and alleviating income inequality. Regarding technological innovation, Schumpeter’s innovation theory points out that innovation is the key driving force for economic growth, including incremental innovation and breakthrough innovation^16^. Digitization provides a platform for countries to learn and communicate, promoting the development of incremental and breakthrough innovations in various countries. Technological innovation enhances solar and wind energy management systems^17^, boosting CP. Regarding alleviating income inequality, digitization can provide job opportunities in low-income areas, enabling participation in carbon emission reduction policy development and implementation^18^. Studying the impact of digitization on CP and its mechanisms is crucial for global efforts in seizing digital opportunities and achieving carbon emission reduction. However, few studies have conducted theoretical analysis and quantitative research on the relationship between digitalization and carbon emissions from an international perspective.

Based on this, this study innovatively starts from an international perspective and incorporates digitization and carbon emissions into a unified analytical framework. First, using the panel data of 136 countries from 2000 to 2020, this study empirically analyzes the impact of digitization on CP and its transmission channels from an international perspective for the first time. Second, using the panel quantile model, this study explores the different effects of digitization on CP at different CP levels. Third, the world sample is divided into Europe, America, Africa, Asia and Oceania according to geographical distribution differences, divided into high income level, middle income level and low income level countries according to income level differences, and divided into high human capital level, middle human capital level and low human capital level countries according to human capital level differences. This study deeply examines the heterogeneous effects of digitization on CP in countries with different locations, income levels and human capital levels. This study provides a new perspective for solving the global carbon emission problem.

The remainder of this study is structured as follows: The second part consists of a literature review. The third part delves into mechanism analysis and hypothesis formulation. The fourth part covers the research area, model, and data. The fifth section presents the empirical results. In the sixth part, we engage in discussion. Lastly, the seventh section concludes with policy recommendations.

## Literature review

In response to global warming, scholars have begun to study CP. In general, CP refers to the economic output generated by unit carbon emissions^3^. It reflects the coordination between economic development and environmental protection^7^. The research content of CP mainly includes measurement methods and influencing factors. Regarding measurement methods, CP is primarily measured using either single-factor or total-factor methods. Single-factor measurement methods mainly include carbon emissions per unit of GDP^19^, carbon emissions per unit of energy consumption^20^, and GDP per unit of carbon emissions^21^. This measurement approach is characterized by its simplicity in calculation and ease of understanding^22^. Total factor measurement methods include stochastic frontier analysis (SFA)^23^ and data envelopment analysis (DEA)^24,25^. The measurement approach considers the input, expected output, and unexpected output, ensuring more accurate and comprehensive results. Regarding the influencing factors, economic development^26^, foreign direct investment^27,28^, industrial structure^29^, urbanization^30^, technological innovation^31^, transportation^11^, environmental regulation^32^, and carbon trading policy^33^ all have an impact on CP. Notably, factors such as industrial structure upgrades, technological innovations, high-speed rail implementations, environmental regulations, and the introduction of carbon trading policies have been found to bolster CP positively. The relationship between economic development and CP is complex, with studies showing it can both increase^34^ and decrease carbon emissions^35^.

Incorporating digitization into the carbon emission analysis framework, scholars have begun to explore the relationship between digitization and CP from different levels. Some scholars have studied the relationship between digitization and CP based on several countries from the international level^36,37^. However, these studies are often limited to a few specific regions and lack an overall perspective on the impact of global CP. Some scholars have studied the region of a particular country from the regional level^38–41^. However, limited by national boundaries, they cannot fully reflect the impact of digitalization on carbon emissions on a global scale. Some scholars have studied the impact of digitization on CP from the enterprise level^42^. While the above research examines the superficial relationship between digitization and CP, it does not delve deeply into the mechanisms through which digitization influences CP.

To address the limitations of previous research, this study offers the following contributions: First, in terms of theory, this study deeply analyzes the mechanism of digitization on CP. It reveals how digitalization can enhance CP through technological innovation and the alleviation of income inequality. On this basis, the fixed effect model and mediating effect model are used to empirically test the impact of digitization on CP and its transmission channels, providing empirical support for it. Second, in terms of sample selection, this study selects the panel data of 163 countries in the world from 2000 to 2020 as the research sample from an international perspective. It fully considers the differences in resource endowment, economic development and education level of different countries, making the research findings more valuable and reference-worthy. Based on this, this study deeply explores the heterogeneous impact of digitization on CP in different countries, which is helpful for decision makers to formulate relevant digitization policies that align with their specific national conditions.

This research is not only of great significance to academia, but also provides valuable insights to policymakers, businesses and individuals. For policymakers, they can develop more effective digital strategies based on their own national characteristics to achieve global carbon emission reduction goals. For businesses, this study can help them better understand the impact of digitalization on carbon emissions, encouraging companies to use digital technologies to reduce their carbon footprint and enhance their competitiveness. For individuals, this research can increase public awareness of the role of digitalization in environmental protection and help improve public environmental awareness and participation.

## Mechanism analysis and hypothesis

### The direct impact of digitization on CP

**Figure 1:**
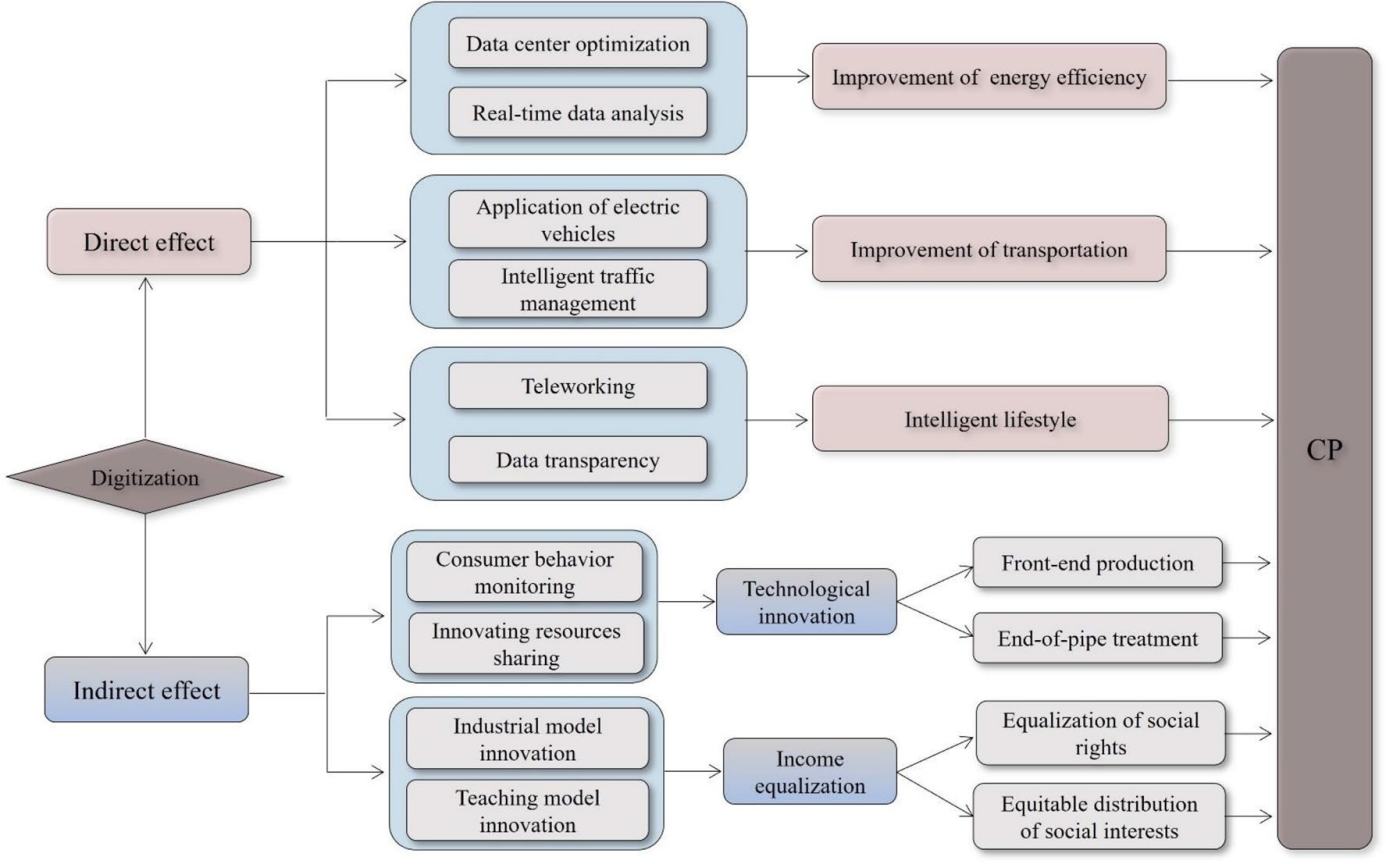
The impact mechanism of digitization on CP.

The direct impact of digitization on CP is mainly reflected in three aspects: Energy efficiency improvement, transportation improvement and lifestyle intelligence (Fig. 1). First, digital technology can monitor the energy use of data centers in real time and optimize their cooling systems. The optimization of cooling systems reduces energy waste^43^ and boosts CP. Digitization enables enterprises to monitor the performance of equipment through real-time data analysis. This allows them to shut down inefficient equipment in a timely manner and improve energy efficiency. Second, digital technology can promote the development of environmentally friendly vehicles such as electric vehicles, thereby reducing carbon emissions^44^. The traffic management system can use vehicle sensors, traffic lights, and other data sources to dynamically adjust the signal timing. Such adjustments mitigate vehicle congestion and cut down on carbon emissions. Third, digital technologies make it possible to work remotely^45^, thereby reducing carbon emissions from commuting. By connecting to applications such as smart watches and traffic tracking systems, people can obtain real-time data on energy consumption and travel modes. Armed with this information, they are encouraged to adopt greener lifestyles. Therefore, Hypothesis 1 is proposed:

#### Hypothesis 1

Digitization can improve CP.

### The indirect impact of digitization on CP

#### Digitization, technological innovation and CP

The impact of digitization on technological innovation is mainly reflected in the following two points: By using digital technologies such as big data and the IoT, enterprises can collect and organize a large amount of user data. With this data, they can precisely pinpoint consumers’ preferences for green products and services. To address the growing green demand of consumers, enterprises increase their R&D investment. This boosts their technological innovation, leading to diverse and innovative green products^46^. Second, Internet platforms like academic databases and professional blogs can effectively integrate global innovation resources, reducing communication and learning costs among countries^47^. Such platforms facilitate the international exchange of innovation resources, further elevating technological innovation.

Technological innovation mainly improves CP through front-end production and end-of-pipe treatment. In front-end production, technological innovation can reduce the cost of utilizing renewable energy sources like solar, wind, and geothermal energy. These renewable sources can serve as viable replacements for traditional coal energy^48^. In addition, advanced energy storage technologies provide the possibility for a continuous supply of renewable energy, making renewable energy widely used in production^49^. In the realm of end-of-pipe treatment, advancements in technologies like carbon capture and utilization facilitate CO_2_ recycling. This not only diminishes CO_2_ emissions, but also produces economic value. Therefore, Hypothesis 2 is proposed:

##### Hypothesis 2

Digitization can improve CP through technological innovation.

#### Digitization, income inequality and CP

The impact of digitization on income inequality is mainly reflected in the following two points: First, with the deepening of digitization, industry and business models continue to innovate, which provides more employment opportunities for low-income groups^18^. In addition, low-income groups can use the network platform to sell handmade products, thereby increasing their income level. Second, the deep integration of digitization and education can elevate the skill level of groups with limited educational resources. This contributes to a reduction in the income disparity. On the one hand, online platforms provide more skills learning opportunities for groups with limited educational resources, including programming and data analysis^50^. The learning of skills enables this group to obtain higher income. On the other hand, using AI and big data, online platforms create personalized learning plans for those with limited education. This boosts their learning efficiency and potential income.

Income equalization increases CP primarily through political and economic channels^51^. Politically, income equality fosters social rights equality. In an environment with equal social rights, individuals access equitable educational and employment opportunities, empowering them to participate actively in decision-making about carbon emission reductions and other environmental policies. Economically, income equality encourages a balanced distribution of social interests, shifting public focus from short-term gains to long-term and global considerations, thereby building a consensus for low-carbon development. Moreover, in an economically equitable society, heightened trust among individuals further solidifies this consensus. Therefore, Hypothesis 3 is proposed.

##### Hypothesis 3

Digitization can improve CP by alleviating income inequality.

## Research area, model and data

### Research area

**Figure 2:**
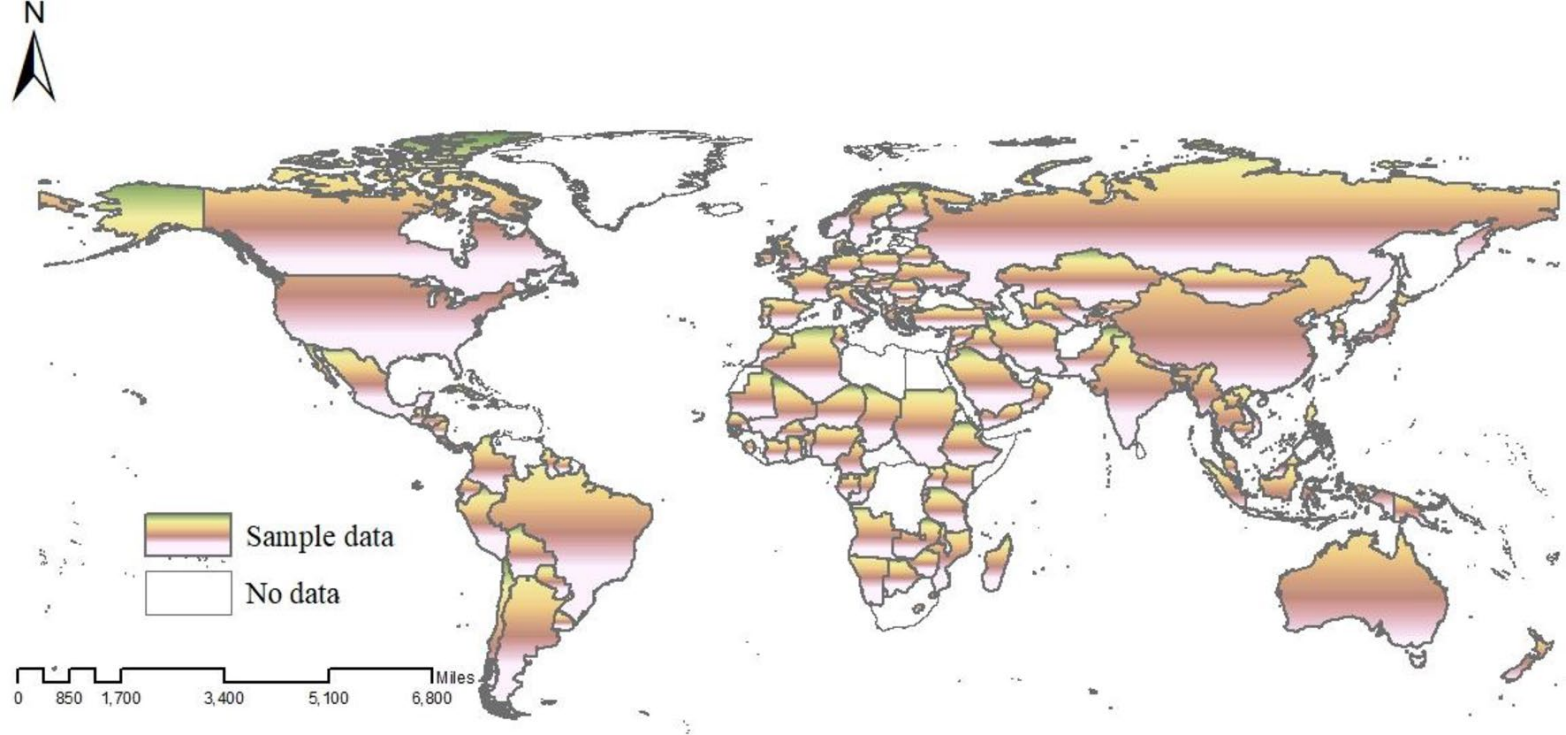
Research area (Map created using ArcGIS 10.2, http://www.esri.com/software/arcgis).

Based on the availability of data, this study selects 136 countries in the world from 2000 to 2020 as research samples. As shown in Fig. 2, these countries span five continents: Europe, Africa, Asia, America, and Oceania, each with its distinct economic and social characteristics. This diversity provides a valuable reference for the heterogeneity analysis in this study.

### Model construction

#### Fixed effect model

In this study, the fixed effect model is employed to empirically examine the impact of digitalization on CP. The fixed effect model is a statistical method used for panel data analysis, taking into account specific characteristics of each observed country, such as economic structure, policy environment, and cultural background. These characteristics remain constant over time but may influence both digitalization and CP. The random effect model and mixed OLS model ignore these characteristics, potentially leading to biased estimation results. The following fixed effect model is constructed:

1 $$\ln CP_it = \alpha_0 + \alpha_1\ln DIG_{it} + \alpha_2ECO_{it} + \alpha_3\ln URB_{it} + \alpha_4\ln POP_{it} + \alpha_5\ln REN_{it} + \lambda_i + \varepsilon_{it}$$

Among them, *lnCP*_*it*_ is the dependent variable representing CP of country *i* in year *t*. *lnDIG*_*it*_ is an explanatory variable representing the degree of digitization of country *i* in year *t*. *ECO*_*it*_, *lnURB*_*it*_, *lnPOP*_*it*_, *lnREN*_*it*_ are the control variables representing economic growth, urbanization level, population density and energy structure of country *i* in year *t*, respectively. $\lambda_i$ is the individual effect. $\varepsilon_it$ is the residual term.

#### Mediating effect model

Furthermore, a three-step stepwise regression method^52^ is used to verify the validity of Hypothesis 2 and Hypothesis 3. The model provides a deeper perspective into the understanding of the complex relationship between digitalization and CP. In addition to Eq. (1), the following two equations should be constructed:

2 $$\ln M_it = \beta_0 + \beta_1\ln DIG_{it} + \beta_2ECO_{it} + \beta_3\ln URB_{it} + \beta_4\ln POP_{it} + \beta_5\ln REN_{it} + \lambda_i + \varepsilon_{it}$$

3 $$\ln CP_{it} = \theta_0 + \theta_1\ln DIG_{it} + \theta_2\ln M_{it} + \theta_3ECO_{it} + \theta_4\ln URB_{it} + \theta_5\ln POP_{it} + \theta_6\ln REN_{it} + \lambda_i + \varepsilon_{it}$$

Among them, *M* represents the mediating variable, which includes technological innovation and income inequality. Equation (2) is used to test the impact of digitization on mediating variables. Equation (3) is used to test the impact of intermediary variables on CP. If $\beta_1$, $\theta_1$ and $\theta_2$ are significant, the mediating variable has a partial mediating effect. The proportion of its mediating effect is $(\beta_1 \times \theta_2)/\alpha_1$. If $\beta_1$ and $\theta_2$ are significant, but $\theta_1$ is not significant, the mediating variable has a complete mediating effect.

#### Panel quantile model

Unlike the fixed effect model. The panel quantile model provides the impact of digitization at different CP quantiles and provides a more comprehensive analysis of heterogeneous features in the data. The following panel quantile model is constructed:

4 $$Q_\tau (\ln CP) = \rho_0 + \rho_1\ln DIG_{it} + \rho_2ECO_{it} + \rho_3\ln URB_{it} + \rho_4\ln POP_{it} + \rho_5\ln REN_{it} + \lambda_i + \varepsilon_{it}$$

Among them, $\tau$ is the quantile value. According to the existing research^53^, this study selects 10%, 25%, 50%, 75% and 90% as the quantile values.

### Variables selection

#### Dependent variable

*Carbon productivity.* The main indicators of CP are CO_2_ emissions per unit of GDP^54^, CO_2_ emissions per unit of energy consumption^20^, and GDP per unit of CO_2_ emissions^21^. Each of these measures has advantages and disadvantages. Regarding carbon dioxide emissions per unit of GDP, this indicator reflects the environmental impact of economic output and is intuitive and easy to understand. Regarding CO_2_ emissions per unit of energy consumed, this measure emphasizes the link between energy use and carbon emissions and is suitable for analyzing carbon emissions in energy-intensive industries. However, it cannot fully reflect the relationship between the economy and environment. Regarding the GDP generated by unit carbon emissions, this indicator reflects the economic output under a certain environmental cost and is also easy for readers to understand. In this study, GDP per unit of CO_2_ emissions is selected as a measure of CP. The reasons are as follows: First, compared with carbon dioxide emissions per unit of GDP, this measurement index focuses more on how to improve economic benefits while achieving low carbon emissions, reflecting a balance between economic growth and environmental protection. Second, this indicator provides an intuitive quantitative method for assessing a country’s economic output under certain environmental costs. Third, GDP is a widely used indicator of economic activity in countries around the world. Using this metric to measure CP allows for comparison and analysis on a global scale.

#### Independent variable

*Digitization.* The popularity of the Internet serves as the foundation for digital application and penetration^55^. Therefore, following the approaches of Koutroumpis^56^ and Yin et al.^57^, this study chooses the proportion of Internet users to the total population as a measure of the level of digitization. The specific reasons are as follows: First, Internet penetration rate can represent the broader digitalization process, including e-commerce, online education, remote work and other aspects. Second, the Internet is one of the digital infrastructures, and its penetration rate directly affects the application and development of digital technology. Third, the Internet penetration rate can directly reflect the basic situation of a country or region’s digitalization level. And this indicator data is easy to obtain. In addition, the mobile phone penetration rate is used as a proxy variable for robustness testing.

#### Mediating variable

*Technological innovation.* Following the approaches of Nguyen et al.^58^, this study selects the proportion of R&D expenditure to GDP as a measure of technological innovation. R&D expenditure is one of the most direct and important inputs to technological innovation. This measure can directly reflect a country or region's investment in scientific and technological research and the development of new products and processes. At the same time, it indirectly reflects the importance a country or region attaches to the knowledge economy.

*Income inequality.* Considering data integrity, this study selects the share of total pre-tax national income held by the top 10% as a measure^18^. This indicator measures the share of overall income earned by the richest small group in society, thereby reflecting the concentration of income at the top of society. Higher values usually mean that income is unevenly distributed and wealth is concentrated in the hands of a few.

#### Control variable

It mainly includes *economic growth*, expressed by the economic growth rate of each country^1^. Swift economic expansion leads to high energy consumption, leading to a substantial production of CO_2_. *Urbanization*, expressed as the proportion of urban population to the total population^59^. The higher the proportion of urban population, the greater the infrastructure construction and energy demand^60^, thus affecting CP. *Population density,* expressed by the number of people per kilometer of land area^61^. The increase of population density increases traffic pressure, thus affecting energy consumption and CO_2_ emissions. *Renewable energy consumption*, expressed by the proportion of renewable energy consumption to total energy consumption^62^. The higher the proportion of renewable energy consumption, the lower the CO_2_ emissions^63^.

### Data sources

This study selects 136 countries from 2000 to 2020 for research. The choice of this period is based on data provided by the World Bank, which has records starting from 2000 and extending to 2022. This period marks a key phase in global digitalization, providing abundant data for researching the relationship between digitalization and CP. When selecting the country sample, this study was limited to 136 countries, mainly because the data are complete in these countries. The lack of key data on digitalization levels and carbon emissions in countries such as Bahrain, Oman, and Guyana limits the scope of research sample. Although these limitations affect the breadth of the sample, the 136 countries selected have covered different economies and geographical regions around the world, ensuring the representativeness and broadness of research results. The data of digitization comes from the International Telecommunication Union database. Income inequality data comes from the income inequality database. Other data are from the World Bank database. In order to eliminate the problem of autocorrelation and heteroscedasticity, this study logarithmically processes all variables except economic growth. The meaning, measurement, unit and source of each variable are shown in Table 1. The descriptive statistics are shown in Table 2.

**Table 1:** Meaning, measurement, unit and source of variables.

Variable	Meaning	Measurement	Unit	Source
CP	Carbon productivity (taking the logarithm)	GDP output per unit of carbon emissions	US$ (constant 2010 US$)/kg	World Bank Database
DIG	Digitalization (taking the logarithm)	Proportion of Internet users	%	World Inequality Database
TI	Technological innovation (taking the logarithm)	R&D expenditure as a percentage of GDP	%	World Bank Database
INC	Income inequality (taking the logarithm)	Pre-tax national income share is held by the top 10% group	%	International Telecommunication Union database
ECO	Economic growth (taking the logarithm)	GDP growth rate	%	World Bank Database
URB	Urbanization (taking the logarithm)	Proportion of urban population to total population	%	World Bank Database
POP	Population density	Number of people per kilometer of land area	The number of people/km^2^	World Bank Database
REN	Renewable energy consumption (taking the logarithm)	Proportion of renewable energy consumption to total energy consumption	%	World Bank Database

**Table 2:** Descriptive statistics of variables.

Variable	Obs	Mean	Std.Dev	Min	Max
lnCP	2856	0.9697	0.7243	− 1.4488	3.0392
lnDIG	2856	2.6319	1.7640	− 8.1481	4.6022
lnTI	2856	− 0.6663	1.2394	− 5.2139	1.5716
lnINC	2856	− 0.7868	0.2016	− 1.3307	− 0.3348
ECO	2856	3.4456	5.1390	− 54.2359	63.3799
lnURB	2856	3.9389	0.4893	2.1097	4.6052
lnPOP	2856	4.1706	1.4315	0.4599	8.9829
lnREN	2856	2.5976	2.2573	− 6.9078	4.5884

## Results

### Spatial–temporal characteristics of digitization and CP

#### Digitization

**Figure 3:**
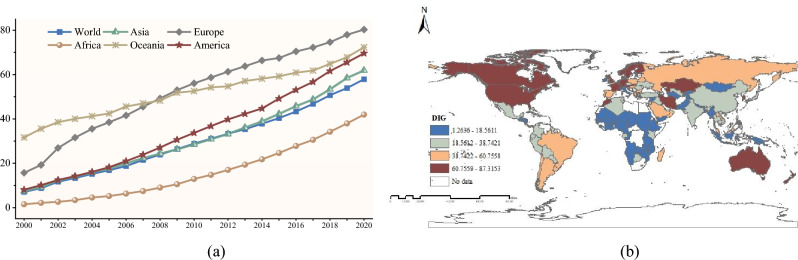
Spatial and temporal characteristics of digitization (Map created using ArcGIS 10.2, http://www.esri.com/software/arcgis).

Figure 3a shows the overall trend of digitization levels from 2000 to 2020, during which the global digitization level increased year by year. This is primarily due to significant investments by many countries in improving Internet infrastructure, leading to continuous digitization enhancement. In addition, the introduction of relevant national policies has also played a crucial role in improving digitization levels. For example, the Chinese government issued the “*National Internet* + *Action Plan*” (2015) and the “*Digital China Construction and Development Strategy*” (2019) to promote digital transformation. In 2017, the UK released the “*UK Digital Strategy*” and in 2016, the German federal government released the “*Digital Strategy 2025*”.

Figure 3b shows the spatial distribution characteristics of digitization. Countries with high levels of digitization are mainly distributed in Europe. European countries have strong economic strength and a lot of resources to improve digital infrastructure and develop digital technology. Meanwhile, the superior education system of European countries also provides lots of professional talents for digital development. Countries with low levels of digitization are mainly distributed in Africa. The economy of African countries is backward, which limits the investment of governments and enterprises in digitalization. In addition, the lack of digital infrastructure such as high-speed Internet connectivity and power supply in African countries is not conducive to the development of digital technology.

#### CP

**Figure 4:**
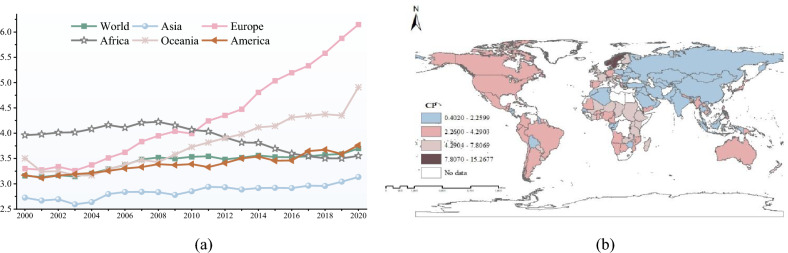
Spatial and temporal characteristics of CP (Map created using ArcGIS 10.2, http://www.esri.com/software/arcgis).

Figure 4a shows the overall trend of CP from 2000 to 2020. Except for African countries, the CP of other countries shows a fluctuating upward trend. In the long run, global carbon emissions have improved. In recent years, the issue of carbon emissions has attracted the attention of countries around the world. Countries have begun to actively develop clean energy and emission reduction technologies to reduce carbon dioxide emissions. After 2008, CP in many countries shows a brief downward trend, which is related to the global economic crisis. After the global economic crisis in 2008, many countries rely on industry to develop their economies and undertake numerous infrastructure projects, consuming significant fossil energy and increasing CO_2_ emissions.

Figure 4b shows the spatial distribution characteristics of CP. Countries with high CP are mainly distributed in Europe. Europe mainly tends to the development of high-tech industries and low-carbon industries, which produce less CO_2_. Countries with low CP are mainly distributed in Asia. These countries rely mainly on extensive production to develop their economies, which consumes a lot of energy and produces substantial CO_2_.

### Preliminary test results

#### Multicollinearity test results

Considering that the regression results of the model can be affected by the correlation of variables, this paper conducts a multicollinearity test. The results are shown in Table 3. The VIF of all variables and their average values are less than 10, indicating that there is no multicollinearity between variables.

**Table 3:** Multicollinearity test.

	VIF	1/VIF
lnDIG	1.77	0.566
ECO	1.03	0.974
lnURB	2.04	0.491
lnPOP	1.05	0.956
lnREN	1.29	0.777
Mean VIF	1.43	

#### Serial correlation test results

In panel data, there may be correlations between data at different time points for the same cross-sectional unit. This article uses Wooldridge to test whether there is time series autocorrelation in the data. The results are shown in Table 4. The P value is less than 0.01, indicating the existence of serial correlation in panel data. Therefore, in the empirical analysis, this paper uses estimation techniques that allow for the existence of serial correlation.

**Table 4:** Serial correlation test.

Wooldridge test	Statistics	Prob	Conclusions
	294.680	0.000	There is serial correlation

#### Cross-sectional correlation test results

Considering the potential correlation between different cross-sectional units, ignoring it may lead to significant estimation biases^64^. This study conducts the Pesaran CD test. Table 5 indicates that there is cross-sectional dependence among countries worldwide. Therefore, in further analyses using this panel data, the study uses estimation techniques that allow for cross-sectional dependence^65^.

**Table 5:** Cross-sectional correlation test.

Pesaran CD test	Statistics	Prob
lnCP	47.88	0.000
lnDIG	413.10	0.000
ECO	141.13	0.000
lnURB	157.32	0.000
lnPOP	282.26	0.000
lnREN	255.23	0.000

#### Panel unit root test results

Considering that the data of each variable may have stationary problems, this paper conducts a Cross-sectional Im, Pesaran, and Shin (CIPS) panel unit root test^66^. Table 6 shows that the first-order difference terms of all variables pass the CIPS test, which verifies the stationarity of variable data^67^.

**Table 6:** CIPS unit root test.

	Level	First-dif	
lnCP	− 2.634*	− 4.298***	Stationary
lnDIG	− 2.607*	− 4.398***	Stationary
ECO	− 3.426***	− 5.227***	Stationary
lnURB	− 1.820	− 2.793**	Stationary
lnPOP	− 1.774	− 2.825**	Stationary
lnREN	− 1.945	− 4.195***	Stationary

#### Panel cointegration test results

Table 7 shows the estimated results of the Pedroni and Westerlund cointegration tests. Both tests reject the null hypothesis of non-cointegration at the 1% level, which indicates that digitization, economic growth, urbanization level, population density and renewable energy consumption have a long-term and stable correlation with CP. Follow-up regression analysis can be performed.

**Table 7:** Cointegration test.

	Pedroni test		Westerlund test	
	Statistic	*P* value	Statistic	*P* value
Modified Phillips–Perron t	9.247	0.000		
Phillips–Perron t	− 7.232	0.000		
Augmented Dickey–Fuller t	− 7.927	0.000		
Variance ratio			− 3.366	0.000

### Benchmark regression results

Table 8 reports the benchmark regression results. The F test shows that the fixed effect model is better than OLS model. Hausman test shows that the fixed effect model is superior to random effect model. Therefore, this study mainly explains the regression results of fixed effect model.

**Table 8:** Benchmark regression results.

	(1)OLS	(2)FE	(3)FE	(4)FE	(5)FE	(6)FE	(7)RE
lnDIG	0.0268***	0.0224***	0.0210***	0.0471***	0.0667***	0.0687***	0.0571***
	(0.0089)	(0.0029)	(0.0030)	(0.0044)	(0.0050)	(0.0049)	(0.0040)
ECO	− 0.0092***		− 0.0019**	− 0.0025***	− 0.0029***	− 0.0027***	− 0.0030***
	(0.0023)		(0.0008)	(0.0008)	(0.0008)	(0.0008)	(0.0007)
lnURB	0.0836**			− 0.652***	− 0.490***	− 0.419***	− 0.435***
	(0.0347)			(0.0795)	(0.0815)	(0.0804)	(0.0613)
lnPOP	0.0917***				− 0.361***	− 0.420***	− 0.146***
	(0.0086)				(0.0474)	(0.0468)	(0.0284)
lnREN	0.216***					0.0786***	0.214***
	(0.0079)					(0.0078)	(0.0096)
Constant	− 0.381***	0.911***	0.921***	3.422***	4.238***	3.993***	2.561***
	(0.147)	(0.0086)	(0.0096)	(0.305)	(0.320)	(0.315)	(0.259)
F test		272.43***	271.33***	269.93***	272.56***	279.72***	
Hausman test		4.66**	4.65*	3.18*	45.72***	98.50***	
LM test							3205.45***
R^2^	0.252	0.121	0.123	0.146	0.166	0.234	0.224
N	2856	2856	2856	2856	2856	2856	2856

Standard errors in parentheses.

***p < 0.01, **p < 0.05, *p < 0.1.

In Column (6), the digital regression coefficient is significantly positive, which verifies Hypothesis 1. Digitization can increase CP. For every 1% increase in digitization level, CP increases by 0.0687%. Regarding the control variables, economic growth, urbanization level and population density all have a negative impact on CP. Economic growth can increase the level of consumption, so that more goods are produced and transported, which consumes a lot of energy and increase carbon emissions. Urbanization advancements boost the need for buildings and infrastructure, leading to higher energy consumption and consequently, increased carbon emissions. The growth in population density means an increase in energy demand, which increases carbon emissions. Renewable energy consumption has a positive impact on CP. The use of renewable energy can replace the application of coal, oil and natural gas in power generation, transportation and industrial production, thereby reducing carbon emissions.

### Robustness test

This study checks the reliability of the estimation results by changing the model and substituting core explanatory variables. Regarding substituting core explanatory variables, this study selects mobile phone penetration rate as a measure of digitization. The estimated results are shown in Column (1) of Table 9. Regarding changing the model, in order to solve the possible endogenous problems between digitization and CP, this study uses the system GMM model to re-estimate the impact of digitization on CP. The estimated results are shown in Column (2) of Table 9. The estimation coefficient of digitization is still significantly positive at the 1% level, which indicates that the benchmark regression results are robust.

**Table 9:** Robustness test results.

	(1) Substituting core explanatory variables	(2) Changing the model
lnDIG	0.0930*** (0.0112)	0.0211*** (0.0027)
ECO	− 0.0047*** (0.0016)	− 0.0081*** (0.0007)
lnURB	− 0.689*** (0.151)	− 0.0449 (0.0289)
lnPOP	− 0.888*** (0.112)	0.0663*** (0.0139)
lnREN	0.368*** (0.0448)	0.123*** (0.0039)
Constant	5.862*** (0.503)	0.529*** (0.116)
R^2^	0.418	
AR(1)		0.000
AR(2)		0.543
Hansen test		1.000
N	2856	2720

Standard errors in parentheses.

***p < 0.01, **p < 0.05, *p < 0.1.

### Mechanism analysis

Table 10 reports the regression results of mediating effect model. Column (1) shows that digitization has a significant role in promoting technological innovation. For every 1% increase in digitization, the level of technological innovation increases by 0.0387%. In Column (3), the estimated coefficient of technological innovation is significantly positive, indicating that technological innovation improves CP. After adding the variable of technological innovation to the model, the estimation coefficient of digitization is reduced, but it is still significantly positive. It shows that technological innovation has a partial mediating effect, and its mediating effect accounts for 22.36% of the total effect. Column (2) shows that digitization has an inhibitory effect on income inequality. Column (4) shows that reduced income inequality can increase CP. Similarly, income inequality also has a partial mediating effect, which accounts for 26.34% of the total effect.

**Table 10:** Mechanism analysis.

	(1) lnTI	(2) lnINC	(3)lnCP	(4) lnCP	(5) lnCP
lnDIG	0.0387*	− 0.0707***	0.0533***	0.0506***	0.0687***
	(0.0213)	(0.0012)	(0.0060)	(0.0049)	(0.0049)
lnTI			0.397***		
			(0.0152)		
lnINC				− 0.256***	
				(0.0779)	
ECO	− 0.0019	0.0004**	− 0.0045***	− 0.0027***	− 0.0027***
	(0.0068)	(0.0002)	(0.0009)	(0.0007)	(0.0008)
lnURB	0.395	− 0.0114	− 0.355***	− 0.417***	− 0.419***
	(0.773)	(0.0202)	(0.105)	(0.0803)	(0.0804)
lnPOP	0.309	− 0.0536***	− 0.445***	− 0.412***	− 0.420***
	(0.436)	(0.0118)	(0.0590)	(0.0470)	(0.0468)
lnREN	0.0303	0.0162***	0.115***	0.0762***	0.0786***
	(0.0736)	(0.0019)	(0.0099)	(0.0079)	(0.0078)
Constant	− 3.685	− 0.551***	3.742***	4.076***	3.993***
	(2.950)	(0.0794)	(0.399)	(0.318)	(0.315)
R^2^	0.162	0.182	0.169	0.103	0.234
N	2856	2856	2856	2856	2856

Standard errors in parentheses.

***p < 0.01, **p < 0.05, *p < 0.1.

### Quantile regression results

Table 11 reports the quantile regression results. With the increase of the quantile level of CP, the regression coefficient of digitization gradually becomes larger, that is, its promotion effect on CP is stronger and stronger. The possible reasons are as follows: First, countries with high CP usually have more environmentally friendly and efficient Internet technologies than countries with low CP, so that digitization plays a greater role in improving CP. Second, with the improvement of CP, people’s awareness of sustainable development and environmental protection increases, which contributes to more efficient application of digital technology. Third, a country with high CP may have a stronger incentive to promote relevant innovation and technological breakthroughs, thereby increasing CP.

**Table 11:** Quantile regression results.

	Q10	Q25	Q50	Q75	Q90
lnDIG	0.0528***	0.0633***	0.0705***	0.0773***	0.0802***
	(0.0083)	(0.0060)	(0.0060)	(0.0089)	(0.0089)
ECO	− 0.0001	− 0.00143**	− 0.0024***	− 0.0036***	− 0.0032***
	(0.0008)	(0.0006)	(0.0005)	(0.0007)	(0.0007)
lnURB	− 0.812***	− 0.580***	− 0.315***	− 0.418**	− 0.263
	(0.156)	(0.0983)	(0.103)	(0.185)	(0.216)
lnPOP	− 0.413***	− 0.423***	− 0.400***	− 0.338***	− 0.296***
	(0.0825)	(0.0783)	(0.0586)	(0.0683)	(0.0866)
lnREN	0.118***	0.148***	0.184***	0.199***	0.127***
	(0.0284)	(0.0228)	(0.0258)	(0.0353)	(0.0418)
Constant	4.739***	3.879***	2.763***	3.062***	2.634***
	(0.618)	(0.447)	(0.379)	(0.689)	(0.831)
R^2^	0.834	0.804	0.783	0.775	0.786
N	2856	2856	2856	2856	2856

Standard errors in parentheses.

***p < 0.01, **p < 0.05, *p < 0.1.

### Heterogeneity analysis

#### Heterogeneity analysis based on different geographical locations

**Figure 5:**
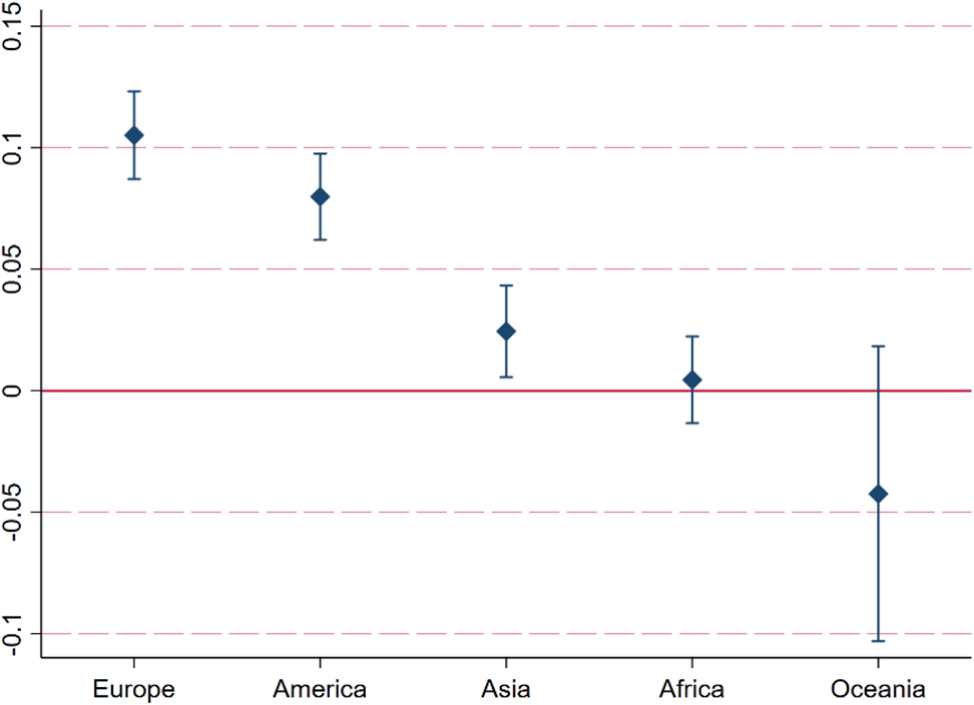
Heterogeneity analysis based on different geographical locations.

In order to explore the differential impact of digitization in different geographical locations on CP, this paper divides the total sample into five subsamples: Europe, America, Asia, Africa and Oceania (Appendix 1). Figure 5 shows the results of heterogeneity analysis based on different geographical locations. The promoting effect of digitization on CP is strongest in European countries. In African countries, the impact of digitization on CP is not significant. In Oceanian countries, digitization has a negative effect on CP. Compared to other countries, European nations have advanced applications in areas such as the IoT, artificial intelligence, and data analysis, which can effectively reduce carbon emissions. Meanwhile, citizens and businesses in European countries are typically more concerned about environmental issues, making them more willing to adopt digital solutions that can reduce carbon emissions. African countries are generally in the early stages of development. Digital infrastructure such as broadband internet and data centers may be relatively underdeveloped, limiting the widespread application and promotion of digital technologies. Therefore, the impact of digitalization on CP is not significant in Africa. Moreover, due to technological limitations and lower energy efficiency, the contribution of digital technologies to improving CP in African countries may be limited, even if some are adopted. In Oceania, although digitalization can improve efficiency in certain industries, the widespread application of digitalization may lead to an overall increase in energy demand, especially in high-energy-consuming technologies such as data centers and cloud computing. This could have a negative impact on the CP of countries in Oceania.

Ma et al.^68^ and Pan et al.^39^ both empirically analyzed the impact of digitalization on China’s carbon emissions using panel data models and found that digitalization has a positive effect on carbon emission performance. This conclusion is different from that of African and Oceanian countries. The main reasons include: The Chinese government has adopted proactive policy measures in promoting the application of digital technologies, which may have accelerated the use of digital technologies in enhancing energy efficiency and optimizing production processes. Compared to some countries in Africa and Oceania, this policy orientation might have led to a more positive impact of digitalization on China’s carbon emission performance. Additionally, China’s efforts in changing its energy structure and increasing the proportion of renewable energy, combined with digitalization, further promoted the improvement of carbon emission performance.

#### Heterogeneity analysis based on different income levels

**Figure 6:**
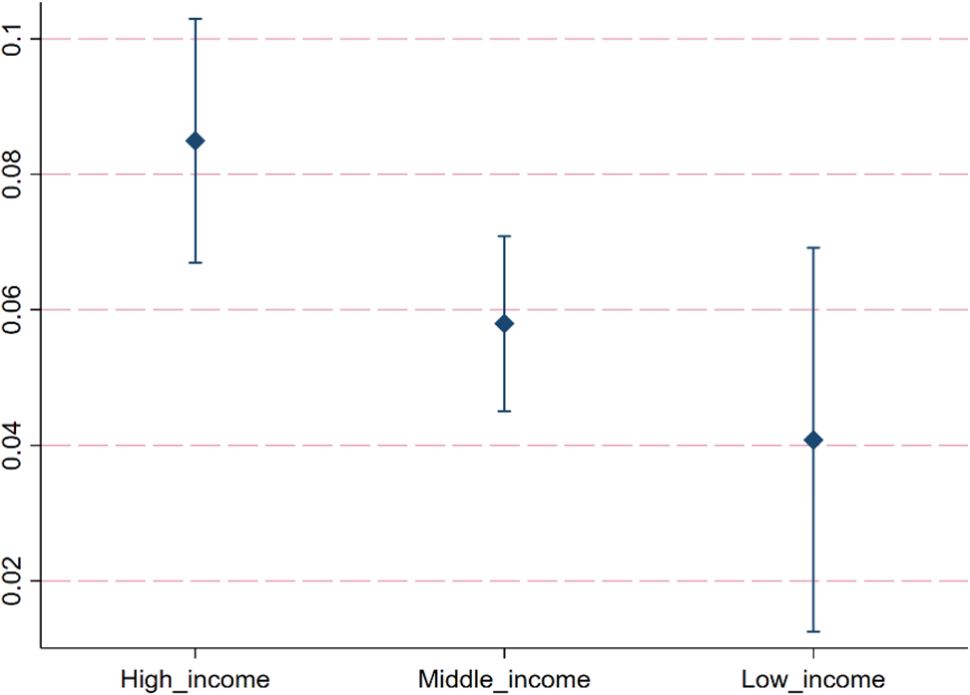
Heterogeneity analysis based on different income levels.

To explore the differential impact of digitization on CP among countries with varying income levels, this study categorizes the overall sample into high-income, middle-income, and low-income countries based on World Bank standards (Appendix 2). Figure 6 shows the results of heterogeneity analysis based on different income levels. The regression coefficients of digitization in high-income, middle-income, and low-income countries are 0.0849, 0.0579, and 0.0408 respectively, all significant at the 1% level. This result is similar to the above results of heterogeneity analysis based on geographical location. Most countries in Europe are high-income. Most in Africa are low-income. The majority of countries in Americas and Asia are middle-income.

Compared to other countries, high-income countries have substantial resources to invest in renewable energy solutions integrated with digital technologies. For instance, they utilize advanced battery technology and the IoT to effectively store and distribute solar and wind energy, thereby enhancing the carbon-reducing effects of digitization. In contrast, low-income countries lack the sufficient financial and technical resources to invest in renewable energy solutions integrated with digitization, which weakens the carbon-reducing effects of digital technology.

#### Heterogeneity analysis based on different human capital levels

**Figure 7:**
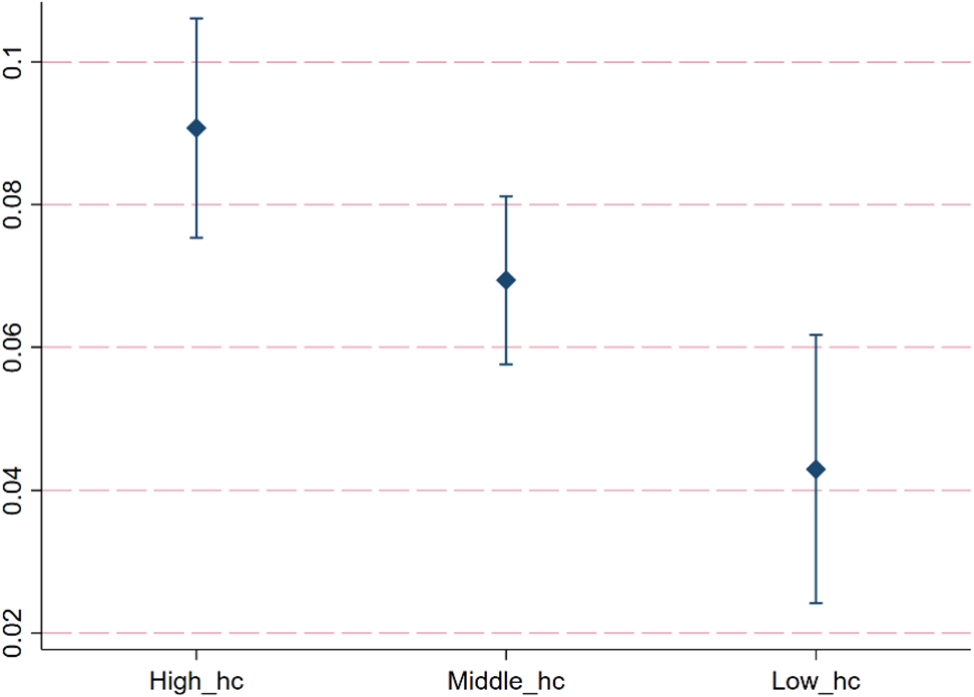
Heterogeneity analysis based on different human capital levels.

To explore the differential impact of digitization on CP among countries with varying levels of human capital, this study divides the overall sample into high human capital countries, middle human capital countries, and low human capital countries (Appendix 3). Referring to the practice of Hao et al.^69^, this paper uses the years of education and the rate of return on education to measure the level of human capital. Data is derives from Penn World Table 10.01. The regression results are shown in Fig. 7. The regression coefficients of digitization in countries with high human capital levels, middle human capital levels, and low human capital levels are 0.0907, 0.0694, and 0.0429, respectively, all significant at the 1% level.

Compared to other countries, countries with high levels of human capital possess a greater number of well-educated engineers and scientists. They can develop and maintain advanced smart grids, IoT, and other digital energy management systems, thereby more effectively managing energy utilization and reducing carbon emissions. Countries with low levels of human capital lack a sufficient pool of professional technical talent, making it challenging for them to develop and maintain complex digital energy management systems.

## Discussion and policy recommendations

Digitization can enhance CP, a finding that aligns with the results of both Liu et al.^70^ and Yang et al.^70^. The reasons are as follows: First, digitization can improve energy efficiency by optimizing data centers and real-time monitoring of equipment performance, thereby increasing CP. Second, digital technology can promote the development of electric vehicles and intelligent traffic management systems, thereby reducing CO_2_ emissions. Third, using digital platforms, citizens can obtain energy consumption-related data, which prompts them to choose a more environmentally friendly lifestyle. Taking Denmark as an example, using the IoT and data analysis, Denmark’s wind power stations can monitor the status of each wind turbine in real time. This allows it to shut down or improve inefficient wind turbines in time, thereby increasing production capacity. In addition, Denmark has introduced a series of policies such as car purchase tax relief to encourage the use of low-carbon transport. Using mobile applications such as smart meters and carbon footprint calculators, Danish citizens can monitor their energy consumption in real time. Consequently, they can assess their carbon impact and choose a more environmentally friendly lifestyle.

Digitization can improve CP through technological innovation. Using digital technologies such as big data and artificial intelligence, enterprises can collect, collate and analyze consumer data. Based on consumer data, enterprises allocate R&D resources to areas with development potential. R&D resources are allocated reasonably, which improves the level of technological innovation of enterprises. In addition, online forums, social media, and specialized blogs offer researchers platforms to share experiences and exchange knowledge. Through these platforms, the flow of technical knowledge is promoted. The global innovation process is accelerated. Technological innovation can improve CP from both front-end production and end-of-pipe treatment. In terms of front-end production, technological innovation mainly improves CP by increasing the use of renewable energy. Through continuous research and development, Germany has achieved significant technological breakthroughs in cutting-edge production equipment, such as efficient solar cells and wind turbines. This has reduced the cost of using renewable energy and promoted its adoption. In terms of end-of-pipe treatment, innovations in technologies such as carbon capture and utilization and waste management can increase CP. At present, Norway has invested in and run several large carbon capture projects that focus on carbon emissions from industrial and electricity production, which increases CP.

Digitization can increase CP by alleviating income inequality. Digitization can provide more employment opportunities for low-income groups through industrial model innovation, such as logistics, sales and customer service, thereby increasing their income. In addition, through innovative educational models, digitization can provide educational resources to groups with limited access, thereby improving their skill levels and incomes. Effectively increasing the income of low-income and educationally disadvantaged groups can help alleviate income inequality. Narrowing the income gap can ensure equal social rights for citizens and provide them the opportunity to participate in shaping carbon emission reduction policies. In New Zealand, a country with a narrow income gap, the government frequently invites the public to contribute to the formulation of carbon emission reduction policies. The primary channels for participation include online platforms, public hearings, and seminars. Moreover, by narrowing the income gap, the New Zealand public tends to prioritize overall interests over private ones, leading to a consensus on low-carbon development.

This study proposes the following policy recommendations:

1. Accelerate the construction of digital infrastructure and enhance the carbon emission reduction effect of digitalization. On one hand, countries around the world should increase their investments in digital technologies such as the IoT, big data, and cloud computing to the extent of their capabilities. Fund the research and development of these digital technologies and support their application across various industries. Combine IoT technology with data analytics to reduce energy consumption in industrial production processes through smart monitoring and control, thereby reducing CO_2_ emissions. On the other hand, countries should use internet platforms to conduct carbon emission reduction training and education. Through activities like online courses and interactive tools, public awareness of environmental protection is enhanced. This encourages more people to adopt actions geared towards energy conservation and emission reduction.
2. Emphasize the inhibitory effect of digitalization on income inequality and promote the deep integration of digitalization with equitable distribution. In terms of job security, digital platforms should be used to promote remote work, especially in low-income areas with fewer traditional employment opportunities. Provide more employment opportunities for low-income areas and disadvantaged groups. In addition, digital platforms can also be used to train job seekers in skills in areas such as digital marketing, programming, and graphic design to enhance their competitiveness. In terms of welfare, the government should use big data and artificial intelligence technology to accurately identify groups that are most in need of social benefits and subsidies. This can be accomplished by analyzing data such as tax records, employment status, educational background, and more. In this way, the government can allocate benefits and subsidies to them more precisely to improve income inequality.
3. Formulate digitalization policies according to local conditions. For economically developed countries, cooling systems and energy management systems should continue to be improved and optimized to reduce energy consumption in data centers, thereby reducing carbon emissions. Encourage data centers to use renewable energy sources such as solar and wind power. Implement energy recovery and reuse strategies, such as using the heat generated by the data center to heat nearby buildings. For economically backward countries, they should actively cooperate with developed countries to obtain the financial and technical support needed to establish digital infrastructure. For example, through international aid or investment by multinational corporations. At the same time, infrastructure such as the Internet is provided to remote areas, as well as basic digital skills training to ensure that people can effectively use these technologies.

## Conclusion

Based on the panel data of 136 countries in the world from 2000 to 2020, this study uses the fixed effect model and mediating effect model to empirically analyze the impact of digitization on CP and its mechanism. Based on different geographical location, income level and human capital level, this study further explores the heterogeneous impact of digitization on CP. In addition, the panel quantile model is also used in this study. The conclusions are as follows: (1) Digitization can effectively improve CP. For every 1% increase in digitization level, CP increases by 0.0687%. (2) From the perspective of impact mechanism, digitization can improve CP through technological innovation and alleviating income inequality. Among them, the mediating effect of technological innovation accounts for 22.36% of the total effect. The mediating effect of income inequality accounts for 26.34% of the total effect. (3) The quantile regression results indicate that as the quantiles of CP increase, the promoting effect of digitalization on CP gradually strengthens. (4) The heterogeneity analysis results show that the impact of digitization on CP is different in countries with different geographical location, income level and human capital level. Regarding geographical location heterogeneity, the promoting effect of digitization on CP is strongest in European countries. In African countries, the impact of digitization on CP is not significant. In Oceanian countries, digitization has a negative effect on CP. Regarding income level heterogeneity, the digitalization in high income countries has the greatest promoting effect on CP, followed by middle income countries, while in low income countries, the promoting effect of digitalization on CP is the least. Regarding human capital level heterogeneity, the digitalization in high human capital countries has the greatest promoting effect on CP, followed by middle human capital countries, while in low human capital countries, the promoting effect of digitalization on CP is the least.

This study explores the impact of digitalization on CP and its mechanism from an international perspective. But there are still some shortcomings. Firstly, this study uses aggregated data of carbon dioxide emissions, which prevents us from observing the impacts of digitalization on different types of carbon emissions such as direct carbon emissions (e.g., car exhaust emissions) and indirect carbon emissions (e.g., emissions from purchased electricity, heat, and steam). In the future, further research could be conducted on this topic to uncover more interesting and meaningful findings. Secondly, this paper innovatively explores how digitalization can enhance CP through technological innovation and the mitigation of income inequality. In addition, digitalization may also improve CP through other means, which leaves some room for discussion in future research.

### Supplementary Information

Supplementary Information.

## Data Availability

The data that support the findings of this study are available from [https://data.worldbank.org/], [https://wid.world/zh/data-cn/].
