# Supplementary material for: Impact of digitization on carbon productivity: an empirical analysis of 136 countries
Source: Sci Rep. 2024 Mar 1;14:5094. doi: 10.1038/s41598-024-55848-2 (PMC10907719; doi:10.1038/s41598-024-55848-2)
Supplement: Supplementary file 1 — Supplementary Information. [file 41598_2024_55848_MOESM1_ESM.docx]

**Appendix 1**

List of sample countries by geographical location

| **Europe** | | **America** | **Asia** | | **Africa** | | **Oceania** |
| --- | --- | --- | --- | --- | --- | --- | --- |
| Albania  Austria  Belarus  Belgium  Bulgaria  Croatia  Denmark  Finland  France  Germany  Greece  Hungary  Ireland  Italy  Luxembourg | Malta  Netherlands  North Macedonia  Norway  Poland  Portugal  Romania  Russia  Slovakia  Spain  Sweden  Switzerland  Ukraine  United Kingdom | Argentina  Belize  Bolivia  Brazil  Canada  Chile  Colombia  Costa Rica  Cuba  Ecuador  El Salvador  Guatemala  Guyana  Honduras  Haiti  Jamaica  Mexico  Nicaragua  Panama  Peru  Paraguay  Suriname  Trinidad and Tobago  Uruguay  United States | Bangladesh  Bahrain  Brunei Darussalam  Bhutan  China  Cyprus  Georgia  Indonesia  India  Iran  Iraq  Jordan  Japan  Kazakhstan  Korea  Kuwait  Laos  Lebanon | Maldives  Myanmar  Mongolia  Malaysia  Nepal  Oman  Pakistan  Philippines  Saudi Arabia  Singapore  Syria  Thailand  Tajikistan  Turkmenistan  Turkey  Uzbekistan  Vietnam  Yemen | Algeria  Angola  Burundi  Benin  Burkina Faso  Botswana  Cote d’Ivoire  Cameroon  Congo  Comoros  Cape Verde  Ethiopia  Equatorial Guinea  Eswatini  Gabon  Ghana  Gambia  Guinea-Bissau  Kenya  Lesotho  Morocco  Madagascar | Mali  Mozambique  Mauritania  Mauritius  Malawi  Namibia  Niger  Nigeria  Rwanda  Sudan  Senegal  Sierra Leone  Seychelles  Chad  Tunisia  Tanzania  Uganda  Zambia  Zimbabwe | Australia  New Zealand  Papua New Guinea |

**Appendix 2**

List of sample countries by income level

| **High-income country** | | **Middle-income country** | | | **Low-income country** |
| --- | --- | --- | --- | --- | --- |
| America  Australia  Austria  Bahrain  Belgium  Brunei Darussalam  Canada  Chile  Croatia  Cyprus  Denmark  Finland  France  Germany  Guyana  Greece  Hungary  Ireland  Italy  Japan  Spain  Korea | Kuwait  Luxembourg  Maldives  Malta  Netherlands  New Zealand  Norway  Oman  Panama  Poland  Portugal  Romania  Saudi Arabia  Seychelles  Singapore  Slovakia  Sweden  Switzerland  Trinidad and Tobago  United Kingdom  Uruguay | Albania  Algeria  Angola  Argentina  Bangladesh  Belarus  Belize  Benin  Bhutan  Bolivia  Botswana  Brazil  Bulgaria  Cambodia  Cameroon  Cape Verde  China  Colombia  Comoros  Costa Rica  Cote d’Ivoire  Cuba  Ecuador  El Salvador  Equatorial Guinea | Eswatini  Gabon  Georgia  Ghana  Guatemala  Haiti  Honduras  India  Indonesia  Iran  Iraq  Jamaica  Jordan  Kazakhstan  Kenya  Kyrgyzstan  Laos  Lebanon  Lesotho  Malaysia  Mauritania  Mauritius  Mexico  Mongolia  Morocco | Namibia  Nepal  Nicaragua  Myanmar  Nigeria  North Macedonia  Pakistan  Papua New Guinea  Paraguay  Peru  Philippines  Russia  Senegal  Suriname  Tajikistan  Tanzania  Thailand  Tunisia  Turkey  Turkmenistan  Ukraine  Uzbekistan  Vietnam  Zambia  Zimbabwe | Burkina Faso  Burundi  Chad  Congo  Ethiopia  Gambia  Guinea-Bissau  Madagascar  Malawi  Mali  Mozambique  Niger  Rwanda  Sierra Leone  Sudan  Syria  Uganda  Yemen |

**Appendix 3**

List of sample countries by human capital level

| **High-human capital country** | **Middle-human capital country** | | **Low-human capital country** | |
| --- | --- | --- | --- | --- |
| Australia  Austria  Belgium  Belize  Bulgaria  Canada  Croatia  Cuba  Denmark  Finland  France  Germany  Hungary  Ireland  Japan  Kazakhstan  Korea  Kyrgyzstan  Luxembourg  Netherlands  New Zealand  Norway  Poland  Romania  Russia  Singapore  Slovakia  Sweden  Switzerland  Tajikistan  Ukraine  United Kingdom  United States | Albania  Algeria  Argentina  Bahrain  Belarus  Bhutan  Bolivia  Botswana  Brazil  Cape Verde  Chad  Chile  China  Colombia  Comoros  Congo  Costa Rica  Cyprus  Ecuador  El Salvador  Gabon  Georgia  Ghana  Greece  Guyana  Honduras  Indonesia  Iran  Iraq  Italy  Jamaica  Jordan | Kenya  Kuwait  Lebanon  Malaysia  Malta  Mauritius  Mexico  Mongolia  Namibia  Nicaragua  North Macedonia  Oman  Panama  Paraguay  Peru  Philippines  Portugal  Saudi Arabia  Spain  Suriname  Syria  Thailand  Trinidad and Tobago  Tunisia  Turkey  Turkmenistan  Uruguay  Uzbekistan  Vietnam  Zambia  Zimbabwe | Angola  Bangladesh  Benin  Brunei Darussalam  Burkina Faso  Burundi  Cambodia  Cameroon  Cote d’Ivoire  Equatorial Guinea  Eswatini  Ethiopia  Gambia  Guatemala  Guinea-Bissau  Haiti  India  Laos  Lesotho  Madagascar | Malawi  Maldives  Mali  Mauritania  Morocco  Mozambique  Myanmar  Nepal  Niger  Nigeria  Pakistan  Papua New Guinea  Rwanda  Senegal  Seychelles  Sierra Leone  Sudan  Tanzania  Uganda  Yemen |
